# PROTOCOL: Digital interventions to reduce social isolation and loneliness in older adults: An evidence and gap map

**DOI:** 10.1002/cl2.1260

**Published:** 2022-06-25

**Authors:** Vivian Welch, Elizabeth Tanjong Ghogomu, Victoria I. Barbeau, Elisabeth Boulton, Sabrina Boutin, Niobe Haitas, Dylan Kneale, Douglas M. Salzwedel, Roger Simard, Paul Herbert, Christopher Mikton

**Affiliations:** ^1^ Methods Centre Bruyere Research Institute Ottawa Canada; ^2^ Division of Nursing, Midwifery and Social Work, School of Health Sciences University of Manchester Manchester UK; ^3^ Canadian Red Cross Montreal Canada; ^4^ Canadian Red Cross Montreal Canada; ^5^ Social Science Research Unit, EPPI‐Centre, UCL Institute of Education University College London London UK; ^6^ Department of Anesthesiology, Pharmacology and Therapeutics University of British Columbia Vancouver Canada; ^7^ Centre Hospitalier de l'Université de Montréal Montreal Canada; ^8^ Department of Social Determinants of Health World Health Organization Geneva Switzerland

## Abstract

This is the protocol for a Campbell systematic review. The objectives are as follows: the aim is to map available evidence on the effects of digital interventions to mitigate social isolation and/or loneliness in older adults in all settings except hospital settings.

## 1 BACKGROUND

### 1.1 Introduction

#### 1.1.1 The problem, condition, or issue

A large body of research shows that social isolation and loneliness are associated with a serious impact on older people's well‐being, mental health, physical health, and longevity (Leigh‐Hunt, 2017; Menec, 2020). Their effect on mortality is comparable to, or even greater, than other well‐established risk factors such as smoking, obesity, and physical inactivity (Holt‐Lunstad, 2015; Ibarra, 2020; Menec, 2020; Windle, 2012).

Social isolation and loneliness are more common in older people and are described as multidimensional concepts with different methods of measurement leading to variations in the prevalence. It ranges from 5% to 43% depending on the study and region (Chen, 2016; Donovan, 2020; Ibarra, 2020; Leigh‐Hunt, 2017). Risk factors include living alone, impaired mobility, experiencing a major life transition change (e.g., loss of spouse or other primary network members), limited income or resources, cognitive impairment, inadequate social support, and geographic location (Cohen‐Mansfield, 2015; Donovan, 2020; Findlay, 2003; Ibarra, 2020).

Although they are related, social isolation and loneliness are two distinct concepts that are often associated with living alone and one may occur without the other. Social isolation is the objective state of lack of interactions with others and the wider community or lack of social relationships (Donovan, 2020; Ibarra, 2020; Leigh‐Hunt, 2017; Menec, 2020). Loneliness is the subjective painful feeling of the absence of a social network or a companion or perception of unmet emotional and social needs resulting from a mismatch between the desired and actual experience of the quality or quantity of social relationships (Cacioppo, 2009, 2014; Menec, 2020; Perlaman, 1981; Prohaska, 2020; WHO, 2021). Therefore, an individual can have a social network and be lonely or a socially isolated individual may not feel lonely. An understanding of the differences in these concepts is important for research in the development of appropriate and effective interventions, and standardizing outcome measurements and also to guide the choice of appropriate interventions for socially isolated or lonely individuals (Fakoya, 2020; WHO, 2021).

Social isolation and loneliness among older people are becoming a priority public health problem and national and international policy issue due to the negative impact on their mental and physical health (Cattan, 2005; Gardiner, 2018; WHO, 2020, 2021). The World Health Organization (WHO) decided, as part of the Decade of Healthy Ageing, to address social isolation and loneliness as a priority issue that cuts across the main action areas of the Decade (WHO, 2020). It is also increasingly being recognized as a public health concern due to the social distancing measures during the COVID‐19 pandemic (Brooke, 2020; Williams, 2021). For example, the average person's daily number of contacts was reduced by up to 74% and almost one quarter of adults in the UK experienced loneliness when living under lockdown (Williams, 2021). Hence the need for digital technology tools to enable remotely delivered interventions to alleviate the impact of social isolation and loneliness during the COVID‐19 restrictions.

There are challenges associated with access to digital interventions and the use of remotely delivered interventions to reduce social isolation and loneliness. Disparities in access to digital interventions and the use of remotely delivered interventions is a growing concern, especially for older adults and during the COVID‐19 restrictions (Budd, 2020; Jopling, 2020; Shah, 2020; Watts, 2020; Williams, 2021). Many older adults lack digital skills and the confidence to access online services and support. Other barriers are affordability and accessibility of technology, broadband or Wi‐Fi, data poverty (i.e., accessibility to wireless Internet connection), geographic divide (rural and urban, high income and low‐ and middle‐income countries). Concerns with digital technology use have also been raised regarding privacy invasion, legal, ethical and clinical data governance during the pandemic through data sharing and access to information (Budd, 2020). Equitable access and support are key in addressing the digital divide.

#### 1.1.2 The intervention

A wide variety of interventions have been developed to reduce social isolation or loneliness among older people. These interventions use different strategies and target different aspects such as facilitating social connections or service provision. They are implemented at different levels such as one‐on‐one or group focused. Although several systematic reviews have evaluated the effectiveness of different types of interventions for social isolation and loneliness in older adults, their findings have sometimes been conflicting (Cattan, 2005; Cohen‐Mansfield, 2015; Dickens, 2011; Findlay, 2003; Gardiner, 2018; Hagan, 2014; Victor, 2018).

Digital interventions have become a particular focus of interest, due partly to the social distancing and lock‐down measures introduced to combat the COVID‐19 pandemic and to the rapidly increasing role technology—particularly the Internet, mobile devices, social media and Internet of things (IoT)—has played in the last 10‐15 years in mediating social relations (Boulton, 2020; Brooke, 2020; Budd, 2020; Falk, 2021; UCLG, 2020; WHO, 2021; Zanella, 2020). They have been used in different sectors (e.g., health care, social services, the community) and in various ways including digital epidemiological surveillance, rapid case identification, interruption of community transmission, public communication, and provision of clinical care and income support and livelihood opportunities in the COVID‐19 crisis.

Digital interventions have also been used to mitigate social isolation and loneliness in older adults by facilitating social interaction or by delivering programs or services (Boulton, 2020; Chen, 2016; Chipps, 2017; Findlay, 2003; Ibarra, 2020; Khosravi, 2016; Noone, 2020; Shah, 2021; Thangavel et al., 2022). They have generally been described as technology‐based interventions to improve communication and social connection among older adults and there is no clear framework for their categorization (Fakoya, 2020). For example, they have been categorized as one‐on‐one or group‐based interventions (Cohen‐Mansfield, 2015; Dickens, 2011; Masi, 2011; Poscia, 2018) or based on four strategies or type (Masi, 2011) as:

- interventions for improving social skills (e.g., computer and Internet training and use with a focus on reducing social isolation or loneliness, online university of the third age);
- interventions for enhancing social support that offer regular contacts, care, or companionship (e.g., telecare with a component to improve social connections, personal reminder information and social management systems (PRISMS), online support groups and forums, social robots or virtual pets, video games, 3D virtual environments, or virtual spaces with trained coaches, conversational agents, or messaging capabilities);
- interventions for enhancing social interaction (videoconferencing, supported video communication, Internet chat facilities, social networking sites, telephone befriending); and
- social cognitive training interventions (low‐intensity psychosocial interventions, Internet‐delivered cognitive behavioral therapy (CBT), mindfulness interventions).

In mapping the body of available evidence, we will categorize interventions by strategies to enable exclusive coding of interventions in categories and subcategories such that an intervention will fit into a single subcategory and not overlap with another on the evidence and gap map.

#### 1.1.3 Why it is important to develop the EGM

Several recent reviews of digital interventions for reducing social isolation and loneliness among older adults indicate there is growing research in this topic area most likely due to the ageing population (Boulton, 2020; Chen, 2016; Chipps, 2017; Ibarra, 2020; Khosravi, 2016; Noone, 2020; Shah, 2021). As well, the COVID‐19 pandemic restrictions have led to a dramatic expansion in the demand for digital technology interventions by people without access including older adults, for the provision of basic services like healthcare, education, and connections with other people (UCLG, 2020). Although there is a very wide range of such interventions, findings on their effectiveness, have sometimes been inconsistent (WHO, 2021). The body of evidence supporting their use is rapidly expanding, dispersed and uneven with lack of consistent terminology. Therefore, the best use of resources at this point for building the evidence architecture needed would be to develop an evidence and gap map on digital interventions to reduce social isolation and loneliness among older people. This evidence and gap map will collate the evidence and display clusters of evidence and gaps in evidence that will serve as a resource to guide prioritization of further research and increase the accessibility and use of evidence for informed decision making by stakeholders including citizens, patients, caregivers, health and social care providers, policy makers and researchers.

#### 1.1.4 Existing EGMs and/or relevant systematic reviews

Recent reviews of digital interventions suggest that (a) there is a very wide range of such interventions; (b) findings on their effectiveness, although sometimes positive, are frequently mixed, inconclusive or uncertain; and (c) the technologies involved are developing rapidly (e.g., artificial intelligence, conversational agents, 3D virtual environments, video‐games, social networking tools) (Boulton, 2020; Chen, 2016; Chipps, 2017; Ibarra, 2020; Khosravi, 2016; Noone, 2020; Shah, 2021).

There is an evidence and gap map on specific remotely delivered interventions (i.e., befriending, social support, and low‐intensity psychosocial interventions) to reduce social isolation and loneliness among older adults (Boulton, 2020). It is based on a rapid review of reviews with systematic review evidence on befriending, social support, and low‐intensity psychosocial interventions that are delivered remotely to older adults excluding caregivers. Study‐level evidence is limited to 18 individual studies in the 5 included systematic reviews.

Our evidence and gap map will be more comprehensive with a broader scope of all types of digitial interventions for older adults including caregivers. It will examine up to date evidence from systematic reviews as well as primary studies and map available evidence to identify gaps and clusters in interventions and outcomes assessed.

## 2 OBJECTIVES

The aim is to map available evidence on the effects of digital interventions to mitigate social isolation and/or loneliness in older adults in all settings except hospital settings.

## 3 METHODS

We will follow the Campbell Collaboration guidance for producing an evidence and gap map (White, 2020).

### 3.1 Evidence and gap map: Definition and purpose

Evidence gap maps are a systematic evidence synthesis product with a visual presentation of existing evidence relevant to a specific research question (Snilstveit, 2013; White, 2020). They display areas with collections or gaps in evidence and the quality of available evidence.

The evidence and gap map is typically a two‐dimensional matrix with interventions as row headings and outcomes as column headings (Snilstveit, 2016; White, 2020). Each cell within the matrix shows the studies with evidence on the corresponding intervention and outcome. This map will identify areas of evidence as well as any gaps in research related to using digital interventions for social isolation and/or loneliness among older adults.

### 3.2 Framework development and scope

We developed an intervention‐outcome framework for this evidence and gap map through a consultative process with stakeholders and adaptation of existing frameworks from systematic reviews, conceptual papers, and reports from stakeholder organizations.

A refined version of the World Health Organization (WHO) Classification of Digital Health Interventions framework (WHO, 2018) was initially considered at the Stakeholder consultation meeting on April 8, 2021. The WHO framework was developed to categorize the different ways in which digital and mobile technologies are used to support healthcare. The stakeholders found the typology of interventions to be too healthcare focused. The consensus was that a more user intuitive typology of interventions was needed to ensure the useability of this evidence and gap map for a larger audience including older adults. A needs‐based approach was preferred as interventions are most effective when they meet the needs and specific circumstances of the older adults (Abdi, 2019; Findlay, 2003; ten Bruggencate, 2019; WHO, 2020).

We identified other relevant frameworks from existing reviews and conceptual papers. We chose two frameworks which used a needs‐based approach (Jopling, 2020) and a strategy‐based approach (Masi, 2011) to address social isolation and loneliness and adapted them for our evidence and gap map.

**Figure 1:**
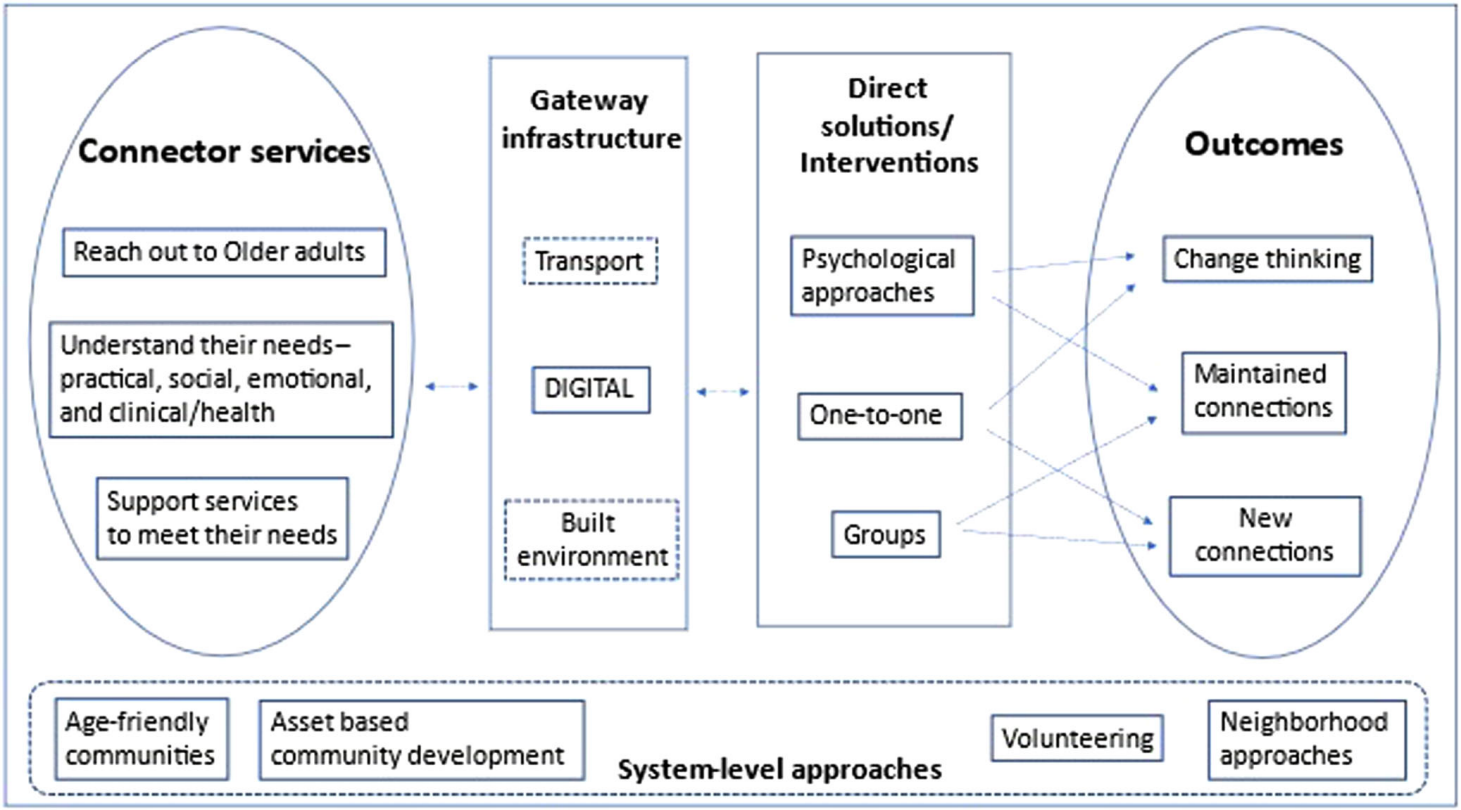
Needs‐based approach framework. Adapted from Jopling (2020).

The needs‐based framework (Jopling, 2020) considers approaches to address loneliness and social isolation that are used in communities to achieve three outcomes: maintain and improve existing relationships or connections, support people to develop new connections, and to change negative thinking about their relationships. The approaches include connector services that reach out to understand the needs of older adults and provide support to meet the needs, gateway infrastructures through which people can connect with others, direct solutions or interventions to reduce loneliness and social isolation, and system‐level approaches that create environments in communities to facilitate tackling loneliness and social isolation (Figure 1).

The intervention categories in this framework do not provide mutually exclusive categorization of digital interventions. For example, many digital interventions such as computer and Internet training, video chats, online cognitive behavioral therapy may be one‐to‐one, or group based. Hence the need for the second framework.

**Figure 2:**
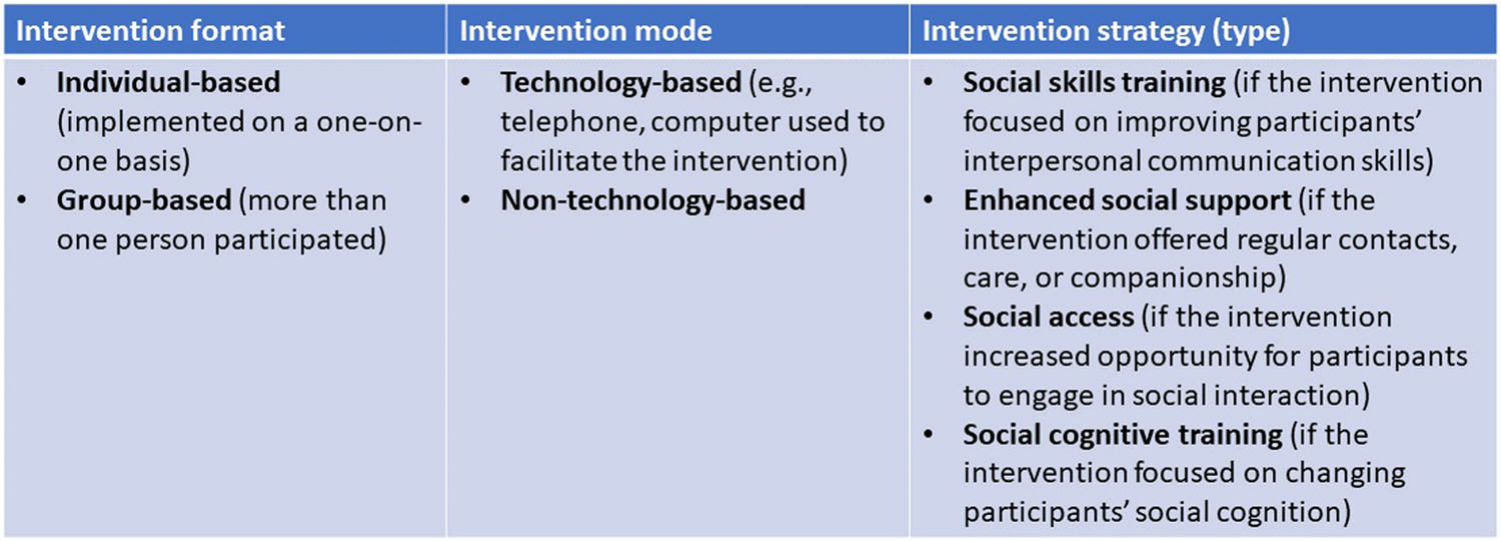
Strategy‐based approach framework. Adapted from Masi (2011).

The strategy‐based model (Masi, 2011) describes strategies used in loneliness reduction interventions based on the understanding of the nature of loneliness and social isolation and how they affect people (Figure 2). Interventions were also categorized based on the format or level of delivery (as one‐on‐one or group interventions) or mode of delivery (technology‐based and non‐technology‐based interventions.

We will use an intervention‐outcome framework where digital interventions of interest will be coded by the strategies to reduce loneliness and social isolation: strategies for (1) improving social skills, (2) enhancing social support, (3) enhancing social interaction, (4) social cognitive training, and (5) multicomponent strategies; as well as by the type of intervention (e.g., computer and Internet training to reduce social isolation and loneliness, video chats, telephone befriending, telecare with a component to improve social connections, online cognitive behavioral therapy). See Supporting Information: Appendix 1 for the glossary of key concepts.

Since the framework is bi‐dimensional (interventions and outcomes), the needs of socially isolated and lonely older adults will be used as a filter on the map and coded interventions will be mapped to the needs.

*Outcomes*: The impacts of interventions to prevent social isolation and loneliness have been measured at different levels—individual, community or societal, and process and implementation levels (Windle, 2012). In our framework, we will consider outcomes that have been identified as indicators of social connection and they will be categorized based on the impact and level of influence of the interventions:

- individual outcomes—loneliness, social isolation, social connectedness, quality of life, anxiety/depression, confidence level, information, communication and technologies (ICT) knowledge and experience, adverse effects;
- community outcomes—social support, social engagement, social cohesion, social capital, digital divide; and
- process indicators—acceptance, adherence, technology use, feasibility, affordability, cost‐effectiveness, barriers.

### 3.3 Stakeholder engagement

We convened an advisory board of 10‐20 stakeholders from organizations such as the International Red Cross, Canadian Red Cross, Agewell, Canadian Frailty Network, HelpAge, CanAge, Centre for Ageing Better, United Nation Department of Social and Economic Affairs, United Nations Fund for Population Activities (UNFPA), and the World Health Organization (WHO). The group of stakeholders includes representatives of these key organizations, policymakers, and academics with an interest in mitigating social isolation and loneliness in older adults. The advisory board provided comments on the intervention‐outcome framework. The WHO Classification of Digital Health Interventions framework was considered. Stakeholders suggested a simplified framework to fit the purpose of this evidence and gap map. The framework was revised, and stakeholders were consulted by email for their feedback on the revised framework included in this protocol.

We consulted with four citizens in two citizen focus groups between June and August 2021. Some iterations were suggested, that is, coding for interventions related to the need of finding purpose in later life, and capturing interventions related to recreation and physical activity. Affordability and access to technology were recommended for consideration in the framework.

### 3.4 Conceptual framework

**Figure 3:**
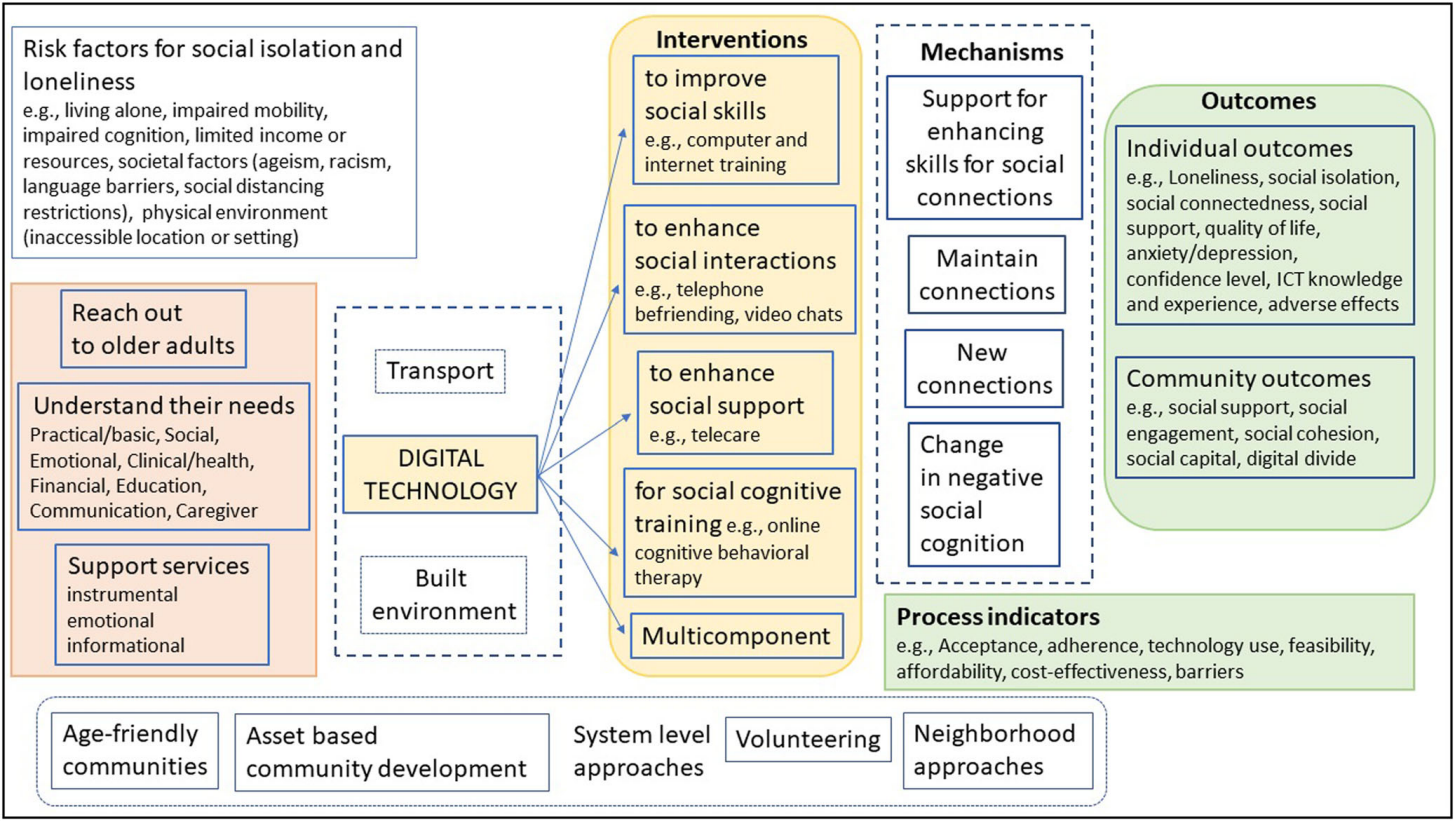
Conceptual framework

Our conceptual framework (Figure 3) is based on the understanding of the needs of older adults, how social isolation and loneliness can occur and how they affect older people's well‐being.

Ageing is associated with a decline in physical and cognitive health, difficulty with mobility, activities of daily living and household routines which put older people at risk of experiencing needs that require health and social support. Social relation is a fundamental part of human nature and social support can be provided through social relations (Abdi, 2019; Tomaka, 2006). Social relation has a structural dimension and a functional dimension (Masi, 2011; Tomaka, 2006; Valtorta, 2016). The structural dimension is defined by the social network ties (number of contacts, frequency of contacts) while the functional dimension is defined by social support including instrumental support (financial, housework, or transportation assistance), informational support (e.g., advice about a purchase or guidance with health systems), emotional support (e.g., expressions of empathy, caring, or trust) and companionship (Menec, 2020; Tomaka, 2006; Valtorta, 2016).

Social isolation and loneliness may be caused by multiple factors and people respond differently depending on their age and coping skills. It is therefore important to reach out to the older adults to understand their circumstances (the risk factors they are facing and their needs) to be able to provide tailored support for social connections or for accessing services as approaches to reduce social isolation and loneliness (Jopling, 2020; ten Bruggencate, 2019).

Support services have been developed to satisfy the needs of older adults and to promote wellbeing and healthy ageing (Abdi, 2019; Jopling, 2020; ten Bruggencate, 2019; WHO, 2020). These needs include social and emotional needs (social connections and companionship), civic engagement (meaningfulness and status, the need for having a purpose in later life or being able to contribute usefully to society), healthcare, housing, home modifications and maintenance, domestic assistance, mobility, nutrition and food security, personal care, education (skills development and learning), financial management, respite care, caregiver support, communication (language support or interpreters, information and assistance/referral services) (Abdi, 2019; Bedney, 2010; Henderson, 2008; Jopling, 2020; WHO, 2020).

Social isolation and loneliness have been associated with low social support and a disruption in social interactions established with other people at any level (individual, group, community, and societal or system) which can lead to unmet needs (Abdi, 2019; Donovan, 2020; Tomaka, 2006). Major changes in life such as change or loss of social network, social participation or role, physical health, mental health and financial resources can also lead to social isolation and loneliness (Donovan, 2020; Newall, 2019; Victor, 2005). Other risk factors for social isolation and loneliness include living alone, societal factors (racism, language barriers, ageism, social distancing and restrictions) and the physical environment (inaccessible location or community setting) (Berkman, 2000; DeGood, 2011; Donovan, 2020).

Different approaches have been used to reduce social isolation and loneliness including facilitating social connections and providing social support. By providing social support services to meet their needs, opportunities for social connections could be created which could reduce social isolation and loneliness in older adults. Support for social connections and companionship or for accessing services can be provided through three gateway infrastructures: digital technology, transportation, or the built environment (Jopling, 2020). We will only consider digital technology. System level approaches to reduce social isolation and loneliness are also beyond the scope of this evidence and gap map.

Based on the understanding of the nature and impact of social isolation and loneliness, different strategies have been used in digital interventions to mitigate social isolation and loneliness: strategies that (1) improve social skills, (2) enhance social interactions, (3) enhance social support, and (4) social cognitive training strategies (Masi, 2011). Since multiple factors may be involved, multicomponent strategies may also be used to address social isolation and loneliness.

The impact of digital interventions has been measured at different levels as individual and community outcomes and process indicators, and they can be achieved by four mechanisms:

1. providing support to building skills for social connections (e.g., computer and Internet training and use, online university of the third age),
2. maintaining existing connections (e.g., video chat with family and friends, personal reminder information and social management system (PRISMS) to engage family and friends in helping receive care, social networking sites),
3. creating new connections (e.g., telephone befriending programs, social networking sites, robots and virtual pets, videogames), and
4. by changing negative social cognition (e.g., online cognitive behavioral therapy to teach lonely people to identify and free themselves from negative thoughts and feelings about their relations such as a perception of lack of intimate attachment to their friends or family).

These mechanisms do not map into the four strategies since some interventions may reduce social isolation or loneliness through more than one mechanism. For example, social networking sites may be used to reduce social isolation and loneliness by maintaining existing connections and by creating new connections. Computer and Internet training can be used to maintain connection with family and friends or to create new connections.

### 3.5 Dimensions

#### 3.5.1 Types of study design

Eligible study designs to be included are completed or on‐going systematic reviews, and primary studies with any form of control group including randomized controlled trials, and evaluative quasi‐experimental designs with a control group.

We will include systematic and scoping reviews based on their PICO question if they explicitly describe adequate search methods used to identify studies, eligibility criteria for selection of included studies, methods of critical appraisal of included studies and synthesis or analysis of included studies (Moher, 2015).

Quasi‐experimental design studies will be eligible if the assignment of participants is based on allocation rules such as alternate assignment (quasi‐randomized studies), inclusion of a threshold on a continuous variable (regression discontinuity designs), or exogenous variation in the treatment allocation (natural experiments) or other rules including self‐selection by investigators or participants, provided data were collected contemporaneously in a comparison group (nonequivalent comparison group design), or an interrupted series design with at least three data points both before and after a discrete intervention (six‐period interrupted time series) (Waddington, 2014).

We will exclude all studies that used less than six period interrupted time series design, or primary studies without a comparison group design like longitudinal cohort studies with no controls, and cross‐sectional studies. We will also exclude literature reviews. However, systematic reviews which also include studies without a comparison group design will be included.

We will not include qualitative research.

#### 3.5.2 Types of intervention/problem

We will consider all types of digital interventions to reduce social isolation and loneliness. These digital interventions may be one‐to‐one, or group based. They may focus on loneliness, social isolation, or both. We will consider any frequency or duration of administration.

We will include the following types of digital interventions categorized by strategies.

- Interventions to improve social skills: these are interventions that focus on training in interpersonal social skills such as conversational skills with the aim to enable individuals to form and maintain meaningful relationships. Examples are computer and Internet training and use to communicate with others, online university of the third age. We will exclude studies that assess computer and Internet training for digital literacy and do not assess the use of Internet to reduce social isolation or loneliness.
- Interventions to enhance social support: these are interventions that offer support (e.g., regular contacts, care, or companionship) and guidance in finding and attending new activities or groups. They aim to help individuals make and maintain social connections. Examples include telecare with a component to improve social connections, personal reminder information and social management systems (PRISMS), online support groups and forums, social robots or virtual pets, video games, 3D virtual environments. We will exclude studies that assess interventions for care without a communication component or a component to improve connecting with other people e.g., smart home technologies like sensors for monitoring falls, e‐health for clinical need only, online cognitive behavioral therapy for dementia care only, online referral systems for healthcare coordination.
- Interventions to enhance social interactions: these are interventions that focus on improving the quality of relationships and increase opportunities for social interactions. They aim to promote connections with family/friends or community and include Internet chat facilities, social networking sites, telephone befriending, for example. Although telephone befrienders could also provide social support, we classify telephone befriending as an intervention to enhance social interactions since the main aim for the service is to connect regularly and build friendship with an older person (Boulton, 2020; Gardiner, 2018).
- Social cognitive training interventions: these are interventions that focus on changing negative thinking and feelings about social relationships. They aim to change behaviors, reduce maladaptive cognitions, and increase social connections. Examples include low intensity psychosocial interventions, Internet‐delivered cognitive behavioral therapy (CBT), mindfulness interventions.

See Table 1 for categories and other examples.

**Table 1:** Intervention categories

Strategy‐based categories and subcategories	Examples
*Interventions to improve social skills*	Training in how to use digital technology—e.g., Computer and Internet training and use Digitally delivered training (e.g., about caregiving/skills building) Digitally delivered learning—e.g., learning a new language, Third age university
Skills development Learning a new activity/language or learning about social skills	
*Interventions to enhance social interaction*	Social connections with family/friends—e.g., video chats Social connections with community—e.g., telephone befriending with volunteers from community
Maintain connections New connections	
*Interventions to enhance social support*	Digital/remote ehealth services—e.g., telecare with a component to improve social connections (HomMed Health Telemonitoring system with a communication component) Digital social and health care coordination with family/friends—e.g., Personal reminder information and social management system (PRISMS) with a communication componentGeolocating/identifying older adults who need services (e.g., Age UK loneliness heat maps) Socially assistive robots (robopets) and virtual pets Virtual spaces Virtual assistants (e.g., Google home, Alexa) Virtual social support groups Digital intergenerational approaches Digital games (e.g., scrabble, chess, cards, exergames) Digitally delivered activities(e.g., exercise—tai chi, yoga,) to mitigate social isolation and loneliness Digital coordination of health or social care services (e.g., online referrals with a component to improve social connections)
Healthcare support Social care support	
*Social cognitive training interventions*	Digital cognitive behavioral therapy Digital mindfulness training Digital psychoeducation Digital reminiscence therapy Digital cognitive behavioral coaching
*Multicomponent interventions*	Including any of the above in a mixed format (e.g., computer training, messaging, and chat groups)

Comparators will be no interventions, other interventions, or usual care.

#### 3.5.3 Types of population (as applicable)

We will include older people, defined as 60 years of age or older (WHO, 2020). If studies include younger and older people, we will include the studies if data can be disaggregated. If data cannot be disaggregated, we will include if the mean age of all participants is at least 65 years of age. To be inclusive, studies or reviews which state a focus on older people without providing the age of participants will be included.

#### 3.5.4 Types of outcome measures (as applicable)

Outcomes will include loneliness, social isolation, as well as other indicators of social connections. Potential harms such as ethical concerns, privacy violations, liability and cyber‐attacks as well as unintended consequences such as increase in social isolation and loneliness, will also be included. Community outcomes such as social support, social engagement, social cohesion, social capital, and digital divide as well as process indicators (acceptability, adherence, technology use, feasibility, affordability, cost‐effectiveness, and barriers), especially for vulnerable populations, will be included (see Table 2).

**Table 2:** Outcome categories

Outcomes	Acceptable measurements
*Individual outcomes*	
Loneliness	UCLA loneliness scale, de Jong‐Gierveld loneliness scale, other scales, e.g., Social and Emotional Loneliness Scale, Hughes loneliness scale
Social isolation	Lubben's Social Network Scale, Social Network Index, PROMIS social isolation 6‐I scale
Social connectedness/interactions/networks or life satisfaction	Lee and Robin's Social Connectedness Scale; Number of contacts; Frequency of social interactions; Satisfaction with interaction; Index of support satisfaction; Support network satisfaction; Companionship scale satisfaction
Social support	Duke‐UNC Functional Social Support Questionnaire; Social support scale by Schuster et al; Hsiung's Social Support Behaviors Scale; Family and Friendship Contacts Scale; Personal Resource Questionnaire; Interpersonal Support Evaluation List (ISEL);
	e‐Diabetes Social Support Scale; a bespoke six‐item scale measuring women's perception of emotional and instrumental support
Well‐being/Quality of life	MOS SF‐36 Health Survey; Work and Social Adjustment Scale (WSAS);
Anxiety/depression	Beck Depression Inventory (BDI); Depression Adjective Check List (DACL) Form E; Geriatric depression scale; The Centre for Epidemiological Studies Depression Scale (CES‐D)
Confidence level	Rosenberg Self‐Esteem Scale
Information, communication and technology (ICT) knowledge and experience	Questionnaire
Adverse effects	Privacy violations, liability, cyber‐attacks, negative effect on well‐being from emotional attachment to devices
*Community outcomes*	
Social support	Duke‐UNC Functional Social Support Questionnaire, Social support scale, social Provisions scale
Social engagement	Engagement in Meaningful Activities Survey (EMAS)
Social cohesion	The Group Cohesion Scale‐Revised; Group Therapy Experience Scale, Group Environment Questionnaire
Social capital	The World Bank's integrated questionnaire for the measurement of social capital (SC‐IQ)
Digital divide	Lack of affordability/access to technology, lack of affordability/access to broadband or Wi‐Fi, data poverty, lack of digital skills or confidence to access services and support online
*Process indicators*	
Acceptability (technology adoption)	
Adherence (training adherence)	
Technology use	Frequency of use
Feasibility	
Affordability	
Cost‐effectiveness	
Barriers	

Outcomes will not be used as eligibility criteria. However, eligible studies and systematic reviews must have a focus on social isolation and loneliness.

#### 3.5.5 Other eligibility criteria

##### Types of location/situation (as applicable)

We will include all country settings as defined by the World Health Organization regions (African Region, Regions of the Americas, South‐East Asian Region, European Region, Eastern Mediterranean Region, Western Pacific Region) (WHO, 2019) and the World Bank classification by income: low income economies, lower‐middle income economies, upper‐middle income economies, high‐income economies (World Bank, 2021).

Primary studies and systematic reviews that do not report the countries will not be excluded.

##### Types of settings (as applicable)

All settings except hospital settings will be included, that is, people living in supportive care institutions (nursing home or long‐term care and assisted living facilities) and in the community (residential or personal home).

### 3.6 Search methods and sources

We designed a search strategy with an information scientist (DS) in consultation with Tomas Allen (WHO information specialist). We will search the following databases from insertion with no date or language restrictions. Ovid MEDLINE, Embase, APA PsycInfo via Ovid, CINAHL via EBSCO, Web of Science via Clarivate, ProQuest (all databases), International Bibliography of the Social Sciences (IBSS) via ProQuest, EBSCO (all databases except CINAHL), Global Index Medicus, and Epistemonikos. The full search strategies are in Supporting Information: Appendix 2.

We will screen reference lists of all included systematic reviews in Eppi‐Reviewer to identify additional studies. We will also contact stakeholders for information about ongoing studies.

### 3.7 Analysis and presentation

#### 3.7.1 Report structure

The report will have the standard sections: abstract, plain language summary, background, methods, results, discussion, and conclusion.

The report will include the flow of studies, included studies, excluded studies and any studies awaiting assessment, as well as synthesis of included studies. We will present the PRISMA flowchart and conceptual framework. We will also include tables and figures that will provide a summary of the distribution of primary studies and systematic reviews across the coding categories such as the type of studies, quality of the systematic reviews, types of interventions, needs, types of populations, outcomes, settings, and geographic distribution.

**Figure 4:**
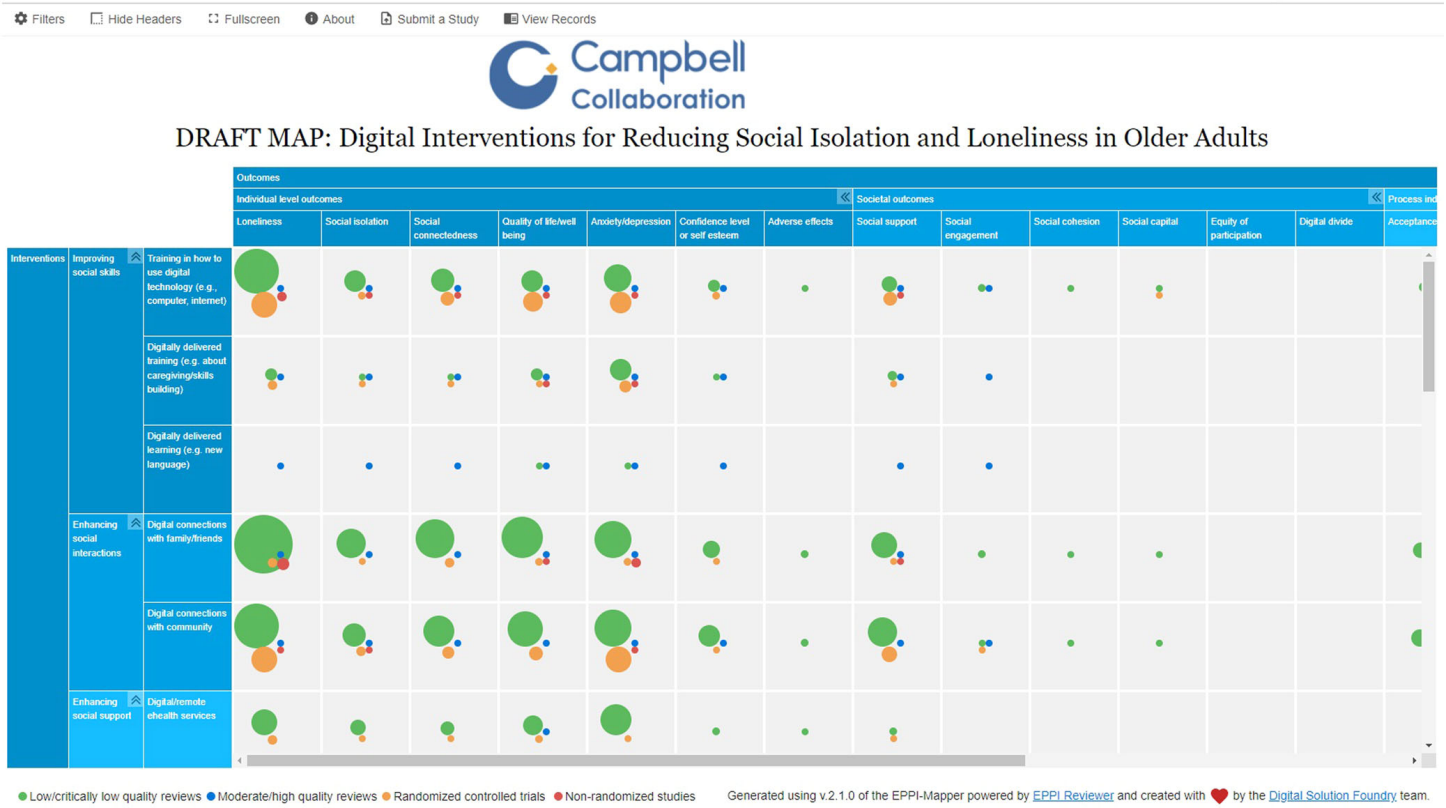
Sample map

The evidence and gap map will have interventions as the row dimension and outcomes as the column dimension. Bubbles of different sizes will represent included studies and different colors will be used to identify the primary studies and methodological quality of the systematic reviews. The filters used in the map will depend on the number of included studies and coded information. See a sample of the map in Figure 4.

#### 3.7.2 Filters for presentation

Additional dimensions of interest that will be used as filters will include the publication status of included studies, study design, World Bank classification by income (low income economies, lower‐middle income economies, upper‐middle income economies, high income economies), and WHO regions (African Region, Regions of the Americas, South‐East Asian Region, European Region, Eastern Mediterranean Region, Western Pacific Region), setting (personal home, independent living/residential home, assisted living, long‐term care/nursing home), health status/condition.

We will document which needs of older adults are being met by digital interventions, using a framework developed from our citizen and stakeholder engagement consultation, which includes social and emotional needs, civic engagement and social participation, healthcare, housing, home modifications and maintenance, domestic assistance, mobility, nutrition and food security, personal care, education, financial management, respite care, caregiver support, communication. We will document the focus of the intervention as aimed at social isolation, loneliness, or both.

##### Equity analysis

We will document whether studies are focused on populations who are at risk or experiencing barriers to health and social care or health inequities across age, sex, ethnicity, income, or other factors. We will use the PROGRESS‐Plus acronym to describe factors associated with health inequities (O'Neill, 2014). For these studies, we will document how potentially vulnerable older people are defined and identified (e.g., using case finding, outreach, screening).

In addition, for each study, we will assess whether studies have analyzed differences in effects for populations experiencing inequities, using the PROGRESS factors (Place of residence (urban/rural), Race/ethnicity/culture and language, Occupation, Gender or sex, Religion, Occupation, Socioeconomic status, Social capital. We will also assess analysis across additional “Plus” factors which are known to be important for older people, including age, health status/condition, frailty, disability, home setting, digital literacy, living situation, social isolation, and loneliness.

#### 3.7.3 Dependency

Multiple reports of the same study will be treated as one study. A study with multiple interventions or outcomes will be shown multiple times on the map (for each intervention or outcome identified). Systematic reviews will be mapped to the interventions and outcomes as defined by the question of the systematic review. Primary studies that meet the eligibility criteria will be mapped as well regardless of whether they are included in one or more systematic reviews.

### 3.8 Data collection and analysis

#### 3.8.1 Screening and study selection

Titles and abstracts and full text of potentially eligible articles will be screened independently following the eligibility criteria in duplicate (by E. G., V. B., P. G., T. H., S. A., N. E., J. E., H. W., and O. D.) using the Eppi‐Reviewer web‐based software program (Thomas, 2020). We will screen systematic reviews based on their PICO questions. Disagreements will be resolved by discussion.

We will use machine learning text mining to support screening at the title and abstract stage. After screening approximately 10% of the titles and abstracts, we will use the priority screening function which develops a classifier based on the probability of inclusion determined from the preliminary screening results. We will, however, double screen all the search results to ensure all potentially eligible studies are captured for the full text screening stage.

We will also screen reference lists of eligible systematic reviews to identify additional studies.

#### 3.8.2 Data extraction and management

We will develop and pilot test a data extraction code set in Eppi‐Reviewer for data collection (see draft in Supporting Information: Appendix 3). After the pilot test, members of the team (E. G., V. B., P. G., T. H., S. A., N. E., J. E., H. W., and O. D.) will individually extract and code data. Automation and text mining will not be used for coding.

The coding categories will include study characteristics (study design, publication status, methodological quality assessment of systematic reviews), intervention categories and subcategories, intervention focus (loneliness, social isolation, or both), outcome domains and subdomains, population characteristics, needs, setting, and location (countries, World Health Organization regions and World Bank classification by income) (Supporting Information: Appendix 4).

We will code description of the population characteristics using the PROGRESS‐Plus framework, defined as Place of residence (urban/rural), Race/ethnicity/culture and language, Occupation, Gender/sex, Religion, Occupation, Socioeconomic status, Social capital (marital status) and additional (plus) factors such as age groups, health status/condition, frailty, disability, home setting, digital literacy, living situation, social isolation, and loneliness.

We will consider how the study population was selected based on whether they are disadvantaged across any PROGRESS‐Plus factors.

We will also code whether there is analysis that aims to understand potential differences across any PROGRESS‐Plus factors.

Given the expected size of the map (of over 200 studies), we will not contact organizations or authors of studies and systematic reviews for missing information.

#### 3.8.3 Tools for assessing risk of bias/study quality of included reviews

We will assess the methodological quality of systematic reviews in duplicate using the AMSTAR 2 tool (Shea, 2017). Any disagreements will be resolved by discussion. As per guidance for evidence maps, primary studies will not be assessed for risk of bias or methodological quality (Snilstveit, 2016; White, 2020).

#### 3.8.4 Methods for mapping

We will use the EPPI‐Mapping tool (Digital Solution Foundry and Eppi‐Mapper, 2020) to develop the evidence and gap map.

## CONTRIBUTIONS OF AUTHORS

Content: PH, CM, VW, EG, RS, SB, NH, EB, DK

EGM methods: VW, EG, VB, EB, DK

Information retrieval: DS

All authors read and approved the protocol.

## DECLARATIONS OF INTEREST

Vivian Welch is editor in chief of the Campbell Collaboration. The editorial process was handled by an independent editor and VW had no input in the editorial process or decisions.

Elisabeth Boulton and Dylan Kneale are joint lead authors of a previous systematic review of systematic reviews which may be eligible for inclusion for the map.

Elizabeth Ghogomu, Victoria Barbeau, Sabrina Boutin, Niobe Haitas, Roger Simard, Paul Hebert, Christopher Mikton have no conflicts of interest.

## PLANS FOR UPDATING THE EGM

The EGM will be updated every 2 years.

## SOURCES OF SUPPORT

Internal sources

• None, Other

External sources

• World Health Organization, Switzerland

WHO funding—Purchase Order Number: 202666968

## Supporting information

Supporting information.
